# Self-Reported Post-COVID-19 Condition and Associated Factors Using Machine Learning Techniques: A Cross-Sectional Study

**DOI:** 10.3390/medicina62091739

**Published:** 2026-09-09

**Authors:** Alaa A. Alghwiri, Ziad Hawamdeh, Abrar F. AlAbed Alhaq, Dania F. Naser, Alia A. Alghwiri

**Affiliations:** 1 Industrial Engineering, German Jordan University, Madaba 11180, Jordan; alaa.alghwiri@gju.edu.jo; 2 School of Medicine, The University of Jordan, Amman 11942, Jordan; z.hawamdeh@ju.edu.jo; 3 Information Systems, Yarmouk University, Irbid 21163, Jordan; abrar.a@yu.edu.jo; 4 Department of Physical Medicine and Rehabilitation, Nobles Rehabilitation Center, Amman 11194, Jordan; dania.naser94@gmail.com; 5 School of Kinesiology, Auburn University, Auburn, AL 36849, USA

**Keywords:** public health, rehabilitation, machine learning, female sex, geriatric, comorbidity, screening

## Abstract

*Background and Objectives*: Post-COVID-19 condition (PCC) is a commonly reported disorder that has gained attention from the World Health Organization (WHO). Several studies have examined factors associated with PCC; however, relatively few have combined statistical and machine-learning approaches. Therefore, this study used statistical analysis to identify factors associated with PCC and machine-learning methods to evaluate the relative importance of these factors and their contributions to model predictions of PCC. *Materials and Methods*: This study employed a cross-sectional observational design in which 963 eligible individuals who had tested positive for COVID-19 were enrolled. Participants were asked about the presence of persistent symptoms lasting for at least 2 months and occurring 3 months after COVID-19 infection, as well as the specific symptoms experienced. The WHO Global COVID-19 Clinical Platform Case Report Form for PCC was used to classify persistent symptoms. Demographic information and medical factors were examined using Poisson regression and machine-learning techniques. *Results*: A total of 209 (22%) out of 963 reported having PCC with fatigue (45%), followed by bone/joint/muscle pain (34%), one neurological symptom (24%), one pulmonary/respiratory symptom (23%), and one mental health symptom (16%) were the most common persistent symptoms. Modified Poisson regression showed that having exactly two chronic conditions, and experiencing two or more previous COVID-19 infections were significantly associated with the prevalence of PCC. The SHAP beeswarm plot indicated that sex, age, time since last COVID-19 infection, number of chronic conditions, and BMI had the greatest influence on the support vector machine (SVM) predictions. Within the fitted model, female sex, age, a longer time since last COVID-19 infection, the presence of chronic conditions, and higher BMI generally shifted predictions toward the PCC category. *Conclusions*: Approximately 22% of participants reported persistent symptoms, with fatigue being the most frequently reported, followed by musculoskeletal pain and symptoms affecting other body systems. In the modified Poisson regression analysis, having exactly two chronic conditions and multiple previous COVID-19 infections were significantly associated with higher prevalence of PCC. However, the machine-learning models demonstrated limited discriminative performance.

## 1. Introduction

Post-COVID-19 condition (PCC), also known as long COVID, is a debilitating disorder that has been raised by the World Health Organization (WHO) in 2022. The WHO has highlighted that the evidence demonstrates the PCC’s severe impact on lives, livelihoods, health systems, and economies [1]. In response, the WHO has been urging researchers and clinicians to implement immediate and sustained measures to address this pressing need [2]. Therefore, the WHO published the Global COVID-19 Clinical Platform Case Report Form (CRF) for post-COVID-19 condition (post-COVID-19 CRF) earlier in 2021 to be used by different countries as a guide for data collection [3].

PCC is a condition that occurs in individuals who were infected with SARS-CoV-2, typically 3 months after the onset of the infection, and have had persistent symptoms for 2 months or more [4]. Fatigue, shortness of breath, joint discomfort, chest pain, cognitive dysfunction, and mental health problems are just a few of the PCC symptoms, which can vary greatly.

The prevalence of PCC symptoms varies greatly among nationalities and countries. Globally, it is estimated that approximately 144.7 million people were living with PCC in 2020 and 2021, highlighting the significant public health burden of PCC [5]. In the U.S., it was found that one in five individuals aged 18–64 years and one in four individuals aged 65 years and older suffer from PCC, highlighting the higher prevalence of PCC in older adults [6].

PCC can have a major effect on people’s quality of life. Some people may experience a protracted disability that impairs their capacity to work or carry out daily tasks. Anxiety and depression may result from weariness and cognitive problems, which can also affect mental health. Furthermore, there is evidence that PCC may increase the risk of contracting additional illnesses, such as diabetes and cardiovascular diseases. A systematic review and meta-analysis of 41 studies involving 860,783 patients identified several risk factors that were significantly associated with developing PCC. These factors included some demographics such as female sex and older age as well as health-related issues such as comorbidities, high body mass index, smoking, and hospitalization [7]. These findings outlined several characteristics associated with an increased risk of developing PCC.

In Jordan, some studies with small sample sizes and/or online surveys investigated the effect of COVID-19 on certain diseases, such as stroke [8]. Two studies investigated specific persistent symptoms after COVID-19 in Jordan, including changes in the menstrual cycle [9] and dizziness or hearing disorders as persistent symptoms [10]. Another study that conducted a survey of 140 medical staff members working at the National Center for Diabetes, Endocrinology, and Genetics in the capital of Jordan reported that about 59% of participants suffered from persistent symptoms [11]. They also indicated that females (79.5%) were more likely than males (20.5%) to have PCC and that fatigue was the most prevalent persistent symptom. An online survey was used in Jordan through social media platforms to identify the prevalence and risk factors of long COVID after only one month of infection [12]. They found that older and obese participants were at lower risk of developing PCC. They also reported that 73.4% of participants had tinnitus, 68.6% reported concentration problems, and 68.3% suffered muscle and joint pain as persistent symptoms [12]. A clinical case-series study on UNRWA staff in Jordan (*n* = 366) reported an incidence of PCC in 20.1% of their staff [13]. Fatigue (39.7%), breathing difficulties with activity (18.8%), amnesia (16.9%), concentrating difficulty (16.7%), anxiety (17.4%), and depression (15.8%) were the most frequently reported persistent symptoms [13]. Another study that included 500 Jordanian participants reported that more than 50% of them suffered from sensory dysfunction and mental health disorders as persistent symptoms after having COVID-19 infection [14]. A recent study that used a web-based questionnaire to assess the prevalence of chemosensory disorders in people with PCC in the Jordanian population found that approximately one-third of the sample suffered from parosmia or parageusia [15].

Effective public health policies and the management of individual health outcomes depend heavily on exploring the burden of PCC and its risk factors. Early identification of patients at higher risk allows for prompt interventions, individualized care, and preventive actions. This insight informs population-level measures to decrease PCC load on healthcare systems and society. This also helps rehabilitation specialists and policymakers take evidence-based decisions and improves their understanding of PCC. However, small sample sizes and inconsistent techniques frequently limit studies on PCC risk factors, which causes reported results to vary. Therefore, the aims of this study were to employ statistical analysis to assess the prevalence of PCC in the capital of Jordan (Amman), investigate the common persistent symptoms after COVID-19 infection, identify factors associated with PCC, and use machine-learning methods to evaluate the relative importance of these factors and their contribution to model prediction of PCC. The findings of this study would provide valuable insights into the prevalence of PCC in Amman. The results would help inform clinical decision-making and improve the quality of life for people with persistent symptoms after COVID-19 infection.

## 2. Materials and Methods

### 2.1. Study Protocol and Ethical Approval

This study employed a cross-sectional observational study. Therefore, it is reported in accordance with the Strengthening the Reporting of Observational Studies in Epidemiology (STROBE) statement, using the checklist specific to cross-sectional studies (Supplementary Material) [16].

Ethical approval was granted by the Institutional Review Board of the Jordan University Hospital (No. 10/2023/10421). To ensure the privacy of participants, every person was assigned a study identification number.

### 2.2. Procedures

A list of names and contact information of adults (18 years or older) who tested positive for COVID-19 infection using PCR test during the year 2022 (1 January 2022 to 31 December 2022) was collected from the information technology department at the Jordan University Hospital, with a total number of 2225 positive cases. Subsequently, a random sample of 1390 individuals was selected from the registry and contacted by telephone. Telephone interviews were conducted between January and May 2023. Among those contacted, 963 eligible individuals provided informed consent and were included in the study (response rate: 69.3%). The remaining individuals were either deceased, ineligible, could not be contacted despite repeated attempts, or declined to participate.

Participants were included if they were aged 18 years or older, were of both sexes, had a confirmed diagnosis of COVID-19 infection at least once, and provided informed consent to participate in the study. Individuals younger than 18 years or those who refused to participate were excluded. Eligible individuals were asked if they had experienced any persistent symptoms lasting for at least 2 months after 3 months of getting COVID-19 infection, consistent with the WHO definition of PCC [4]. To ensure that these persistent symptoms were attributable to COVID-19 rather than to other underlying or pre-existing conditions, participants were specifically asked to report whether the symptoms had newly developed following their COVID-19 infection and whether they could not be explained by any other diagnosed medical condition.

Participants who reported persistent symptoms were asked to identify the specific self-reported symptoms they experienced. To ensure standardized data collection, symptom information was obtained using the WHO Post-COVID-19 Case Report Form (CRF), a validated and internationally standardized instrument developed by the WHO to collect clinical data on the medium- and long-term consequences of COVID-19. The CRF includes a structured symptom checklist, allowing participants to report the presence or absence of a range of post-COVID-19 symptoms in a standardized manner [3]. The post-COVID-19 CRF was used to classify persistent symptoms into the following systems: cardiovascular, endocrine, gastrointestinal, generic fatigue, musculoskeletal, mental health, neurological, pulmonary, and renal [3]. Finally, health-related information of participants was collected from their medical records, including age, sex, marital status, education level, height, weight, number of medications, number of chronic conditions, and number of COVID-19 infections.

### 2.3. Factors Examined in Relation to PCC

The factors that were initially considered in this study comprised demographic characteristics, including age, gender, marital status, and education level, as well as medical characteristics, including body mass index (BMI), number of medications, number of chronic conditions, time since last COVID-19 infection (in months), and total number of confirmed COVID-19 infections. Factors related to COVID-19 symptoms were also examined and included fatigue; bone, joint, or muscle pain; urinary and reproductive symptoms; neurological, pulmonary, cardiovascular, gastrointestinal, mental health, and endocrine symptoms. Because the number of chronic conditions and the number of medications were highly correlated [Variance Inflation Factor (VIF) > 10], the number of medications was excluded from the multivariable model following consultation with domain experts. In addition, COVID-19 symptom categories were not included as explanatory variables because these data were recorded only for participants who met the criteria for PCC. Instead, symptom categories were summarized descriptively in the Results Section to identify the most frequently reported symptoms among participants with PCC.

### 2.4. Statistical Analysis

The goal of this study was to identify the characteristics of those who had PCC. Accordingly, having persistent COVID-19 symptoms was the binary outcome of interest where those individuals with persistent COVID-19 symptoms were labeled 1 and 0 otherwise. Categorical variables were presented as proportions, while continuous variables were summarized using means and standard deviations. The Kolmogorov–Smirnov test was used to assess the normality of continuous variables. For normally distributed variables, we used Student’s *t*-test; otherwise, the Mann–Whitney U test was used. Differences in categorical variables were analyzed using the Chi-squared test. The analysis was conducted using Python (version 3.13.3), with key libraries including Pandas (version: 3.0.2) for data manipulation, Numpy (version: 1.26.4) for numerical operations, Matplotlib (version: 3.10.8) and Seaborn (version: 0.13.2) for visualization, SHAP for factors’ relative importance and the density scatter plot, and scikit-learn for model development and evaluation. R software (version 4.5.2) was also used to generate summary statistics and the modified Poisson regression.

#### 2.4.1. Modified Poisson Regression

A modified Poisson regression approach was selected for the analysis because this was a cross-sectional study and the prevalence of persistent COVID-19 symptoms was approximately 22%, making adjusted prevalence ratios more directly interpretable than odds ratios. The model used a log link and robust variance estimation to evaluate the associations between demographic and medical characteristics as well as the prevalence of persistent symptoms. Model assumptions and adequacy were assessed using diagnostics appropriate for modified Poisson regression, including independence of observations, correct binary outcome coding, and linearity of continuous variables on the log-prevalence scale, absence of severe multicollinearity and highly influential observations, and appropriate use of robust variance estimation. Multicollinearity among the explanatory variables was evaluated using VIFs. The primary modified Poisson regression analysis was conducted using complete cases. To assess the robustness of the findings to missing-data handling, the same model was additionally fitted to the imputed dataset as a sensitivity analysis, and adjusted prevalence ratios, 95% confidence intervals, and *p*-values were compared between the two analyses (Table A1).

#### 2.4.2. Data Preprocessing and Preparation for Machine Learning

Best practices recommended by the ROBUST-ML checklist were followed to design and benchmark our machine learning algorithms [17]. To assess data completeness, the number and proportion of missing observations were examined for each variable. All variables had complete data except age, marital status, education level, BMI, and number of chronic conditions, for which 25%, 28%, 28%, 28%, and 29% of observations were missing, respectively. The pattern of missing values was examined across the study variables before imputation. Because the machine-learning models required complete predictor data, missing categorical predictors were imputed using the mode, whereas missing continuous predictors were imputed using K-nearest neighbors (KNN) with five nearest neighbors. The outcome variable was not imputed. The resulting imputed dataset was used for machine-learning model development and was additionally used in the modified Poisson sensitivity analysis described in Section 2.4.1. Data transformation steps included normalizing continuous variables using the z-score and encoding categorical variables. The data was randomly split into two sets: 80% training (770 patients) and 20% internal testing (193 patients). Stratified sampling was used to maintain the same proportion of patients with persistent COVID-19 symptoms in both the training and testing sets. Since the outcome was not balanced (22% of patients had persistent COVID-19 symptoms), the Synthetic Minority Oversampling Technique (SMOTE) was used to balance the training data to grant each algorithm a chance to learn from each feature equally. The training and testing datasets were cleaned and prepared independently to avoid data leakage.

#### 2.4.3. Model Development, Tuning, Selection, and Validation

Initially, five of the best-performing machine learning classifiers were selected: logistic regression (LR) as a baseline model, random forest (RF), support vector machines (SVM), gradient boosting (GB) and artificial neural network (ANN). To identify the optimal hyperparameters for each algorithm, randomized search cross-validation was performed using 10-fold cross-validation, 10 iterations, and a random state of 42. Regularization techniques were applied to prevent overfitting. After selecting the optimal hyperparameters, models were retrained on the entire training set to derive final weights and create a lockout model to evaluate on the hold-out test set. The five models were then evaluated using the area under the receiver operating characteristic (AUROC) and its associated confidence interval on the hold-out test set to pick the final candidate model. To ensure good generalization performance, the best classifier was chosen by considering the trade-off between bias and variance. Several metrics were reported along with the AUROC for the five models, including accuracy, sensitivity (recall), specificity, positive predictive value (PPV) or precision, negative predictive value (NPV), and F1-score. The ROC-optimized values were chosen using Youden’s index. To understand each predictor’s contribution to the final prediction score, we used SHAP. This game theory-based approach explains the machine-learning model outputs by measuring each predictor’s influence. Finally, a beeswarm plot, generated using SHAP values from the best-performing model, was used to visualized predictor importance and how variations in predictor values impact predictions.

## 3. Results

### 3.1. Participants’ Characteristics and Comparison Between Individuals with and Without PCC

The study sample comprised 963 participants, of whom 209 (22%) reported persistent symptoms meeting the criteria for PCC. Demographic and health-related characteristics for the overall sample (*n* = 963), participants without reported PCC (*n* = 754), and participants with PCC (*n* = 209) are presented in Table 1. In the unadjusted comparisons, statistically significant differences between participants with and without PCC were observed for the number of chronic conditions (*p* = 0.04) and the number of previous COVID-19 infections (*p* < 0.01).

### 3.2. Modified Poisson Regression Results

In the multivariable modified Poisson regression with robust variance (Table 2), participants with exactly two chronic conditions had a significantly higher prevalence of PCC compared with those without chronic conditions (aPR = 1.685; 95% CI: 1.120–2.534; *p* = 0.012). The number of previous COVID-19 infections was also significantly associated with PCC. Compared with participants who had experienced one infection, those with two infections had approximately 88% higher prevalence of PCC (aPR = 1.882; 95% CI: 1.159–3.058; *p* = 0.011), while those with three or more infections had approximately three times the prevalence of PCC (aPR = 3.027; 95% CI: 1.792–5.115; *p* < 0.001). No statistically significant associations were observed for age, sex, marital status, education level, BMI, having one or three or more chronic conditions, or time since the last COVID-19 infection.

### 3.3. Machine-Learning Model Performance and Factors’ Importance

Five classification algorithms (LR, RF, SVM, GB, and ANN) were utilized using the training dataset and evaluated on the independent testing dataset. Hyperparameter tuning was conducted using the training data. The final random forest model used 100 trees, a maximum tree depth of 2, a minimum of 30 observations required to split an internal node, a minimum of 40 observations per terminal node, and log_2_ feature selection. The gradient boosting model used 100 estimators, a learning rate of 0.01, a maximum depth of 3, a subsampling rate of 0.60, a minimum of 50 observations required for splitting, a minimum of 15 observations per terminal node, and square-root feature selection. The final SVM used a second-degree polynomial kernel with C = 0.1 and gamma set to “scale.” The ANN consisted of two hidden layers with 10 neurons each, rectified linear unit activation, the Adam optimizer, an initial learning rate of 0.0003, an L2 regularization parameter of 1.0, and a maximum of 300 iterations.

Classification thresholds were selected by maximizing Youden’s index using out-of-fold predictions generated from the training data and were subsequently applied unchanged to the testing dataset. The selected thresholds were 0.5502 for logistic regression, 0.5228 for random forest, 0.5341 for gradient boosting, 0.4866 for the SVM, and 0.5582 for the ANN.

**Figure 1:**
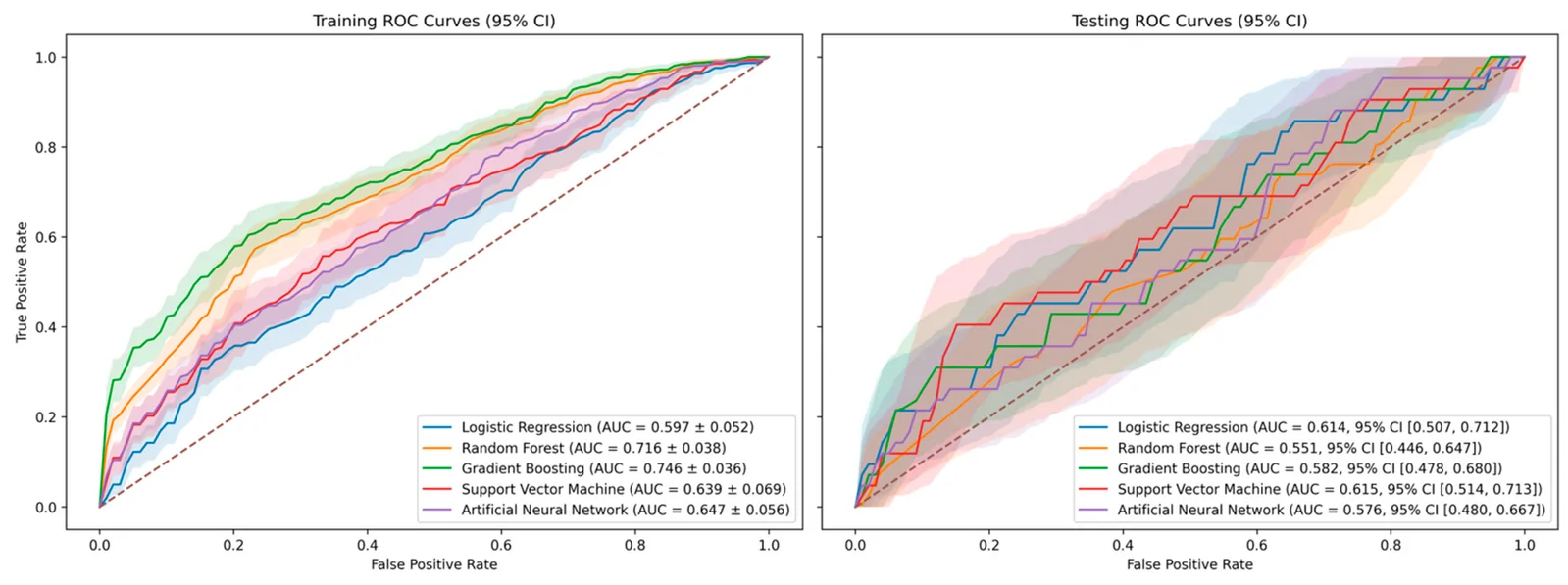
AUROC on the training and testing sets. Receiver operating characteristic (ROC) curves comparing the discriminative performance of the five classification models on the training and independent testing datasets. The diagonal line represents random classification.

Figure 1 compares the AUROC values obtained from the training and testing datasets. Overall, the models demonstrated limited discriminative performance. The SVM achieved the highest testing-set AUROC of 0.615 (95% CI: 0.514–0.713), although this was nearly identical to the AUROC of 0.614 observed for logistic regression. Therefore, the SVM did not demonstrate a meaningful improvement in overall discrimination compared with the conventional regression model. However, at the selected threshold, the SVM achieved a comparatively higher recall, indicating that it identified a greater proportion of participants with PCC. Detailed testing-set performance measures, including accuracy, precision, recall, specificity, positive predictive value, negative predictive value, F1-score, and AUROC, are presented in Table 3.

**Figure 2:**
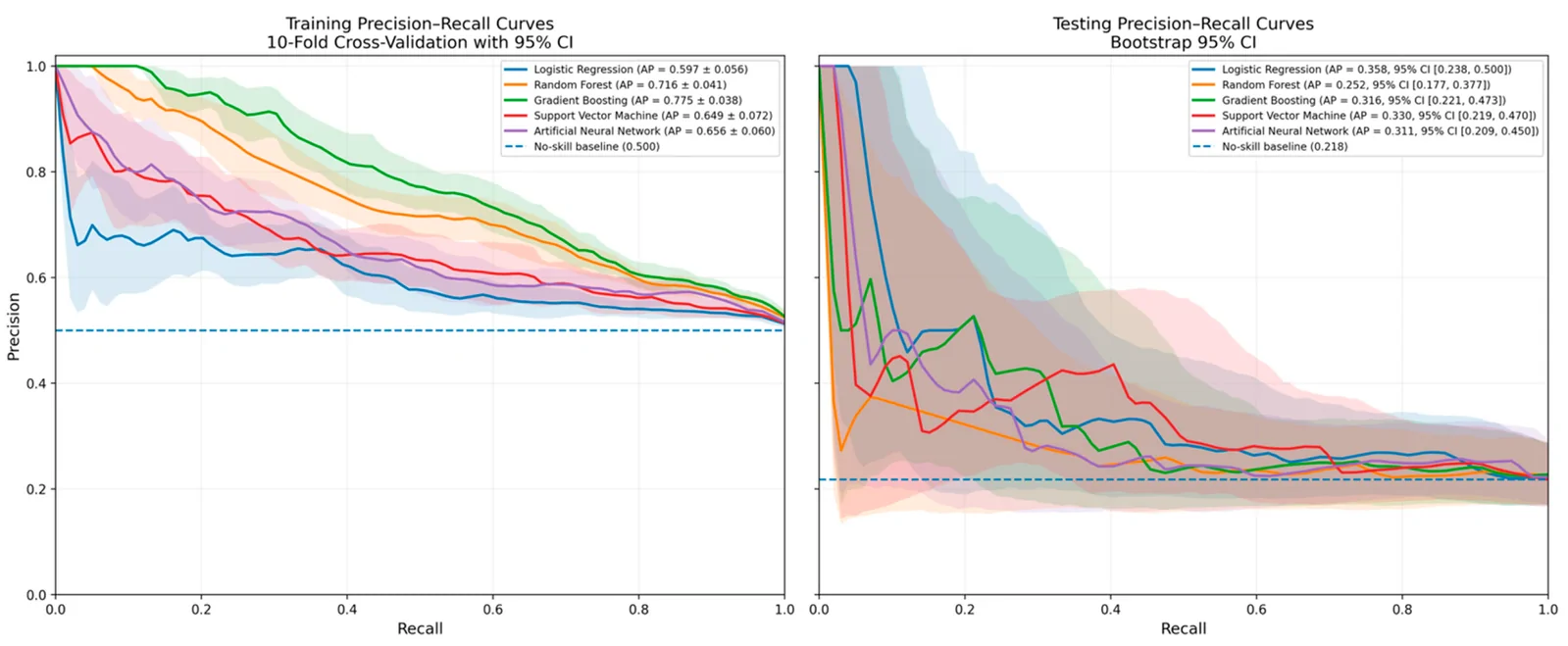
Precision–recall curves for training and testing datasets. Precision–recall curves comparing model performance across training and testing datasets. Higher curves indicate a better balance between precision and recall for identifying participants with PCC.

Figure 2 presents the precision–recall curves for the training and testing datasets. These curves provide additional information regarding each model’s ability to identify participants with PCC, particularly given the imbalance between participants with and without PCC. The precision–recall results were consistent with the AUROC findings and indicated that the models had limited ability to distinguish participants with PCC from those without PCC.

**Figure 3:**
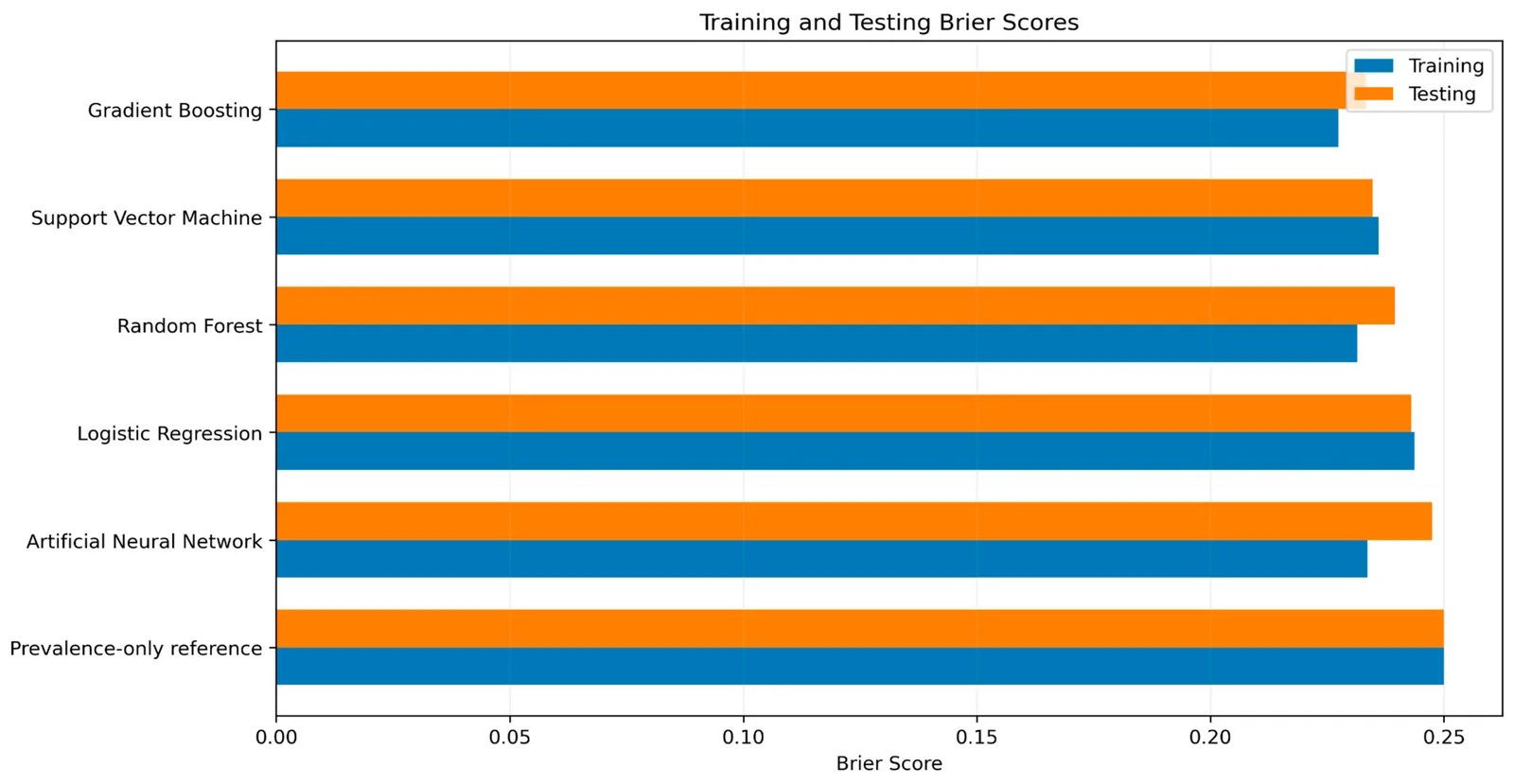
Comparison of Brier score across all algorithms. Training and testing Brier scores for the five classification models. Lower Brier scores indicate better agreement between predicted probabilities and observed PCC outcomes.

Figure 3 compares the training and testing Brier scores across the five classification algorithms. Gradient boosting achieved the lowest testing Brier score, followed closely by the SVM, indicating a comparatively better agreement between predicted probabilities and observed PCC outcomes. Random forest and logistic regression produced slightly higher Brier scores, whereas the ANN had the highest testing score. Nevertheless, the differences among the algorithms were modest. The relatively similar training and testing scores observed for gradient boosting, SVM, and logistic regression suggested limited overfitting, while the larger differences observed for random forest and the ANN indicated somewhat weaker generalization.

**Figure 4:**
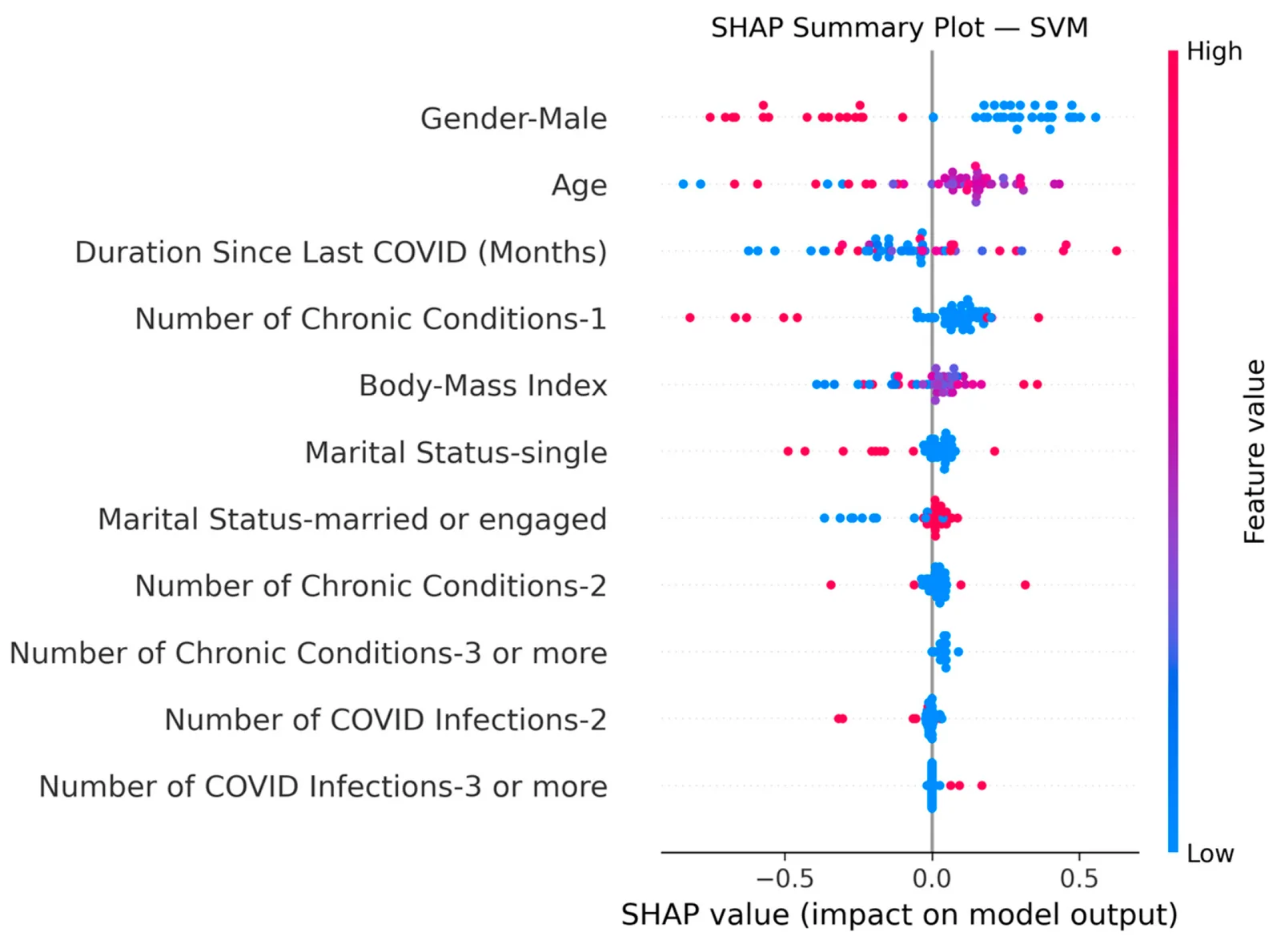
SHAP beeswarm plot for the support vector machine model. Each point represents the SHAP value for an individual participant and feature. Factors are ordered according to their overall influence on model predictions. Positive SHAP values shift the model prediction toward PCC, whereas negative values shift the prediction away from PCC. Feature values are represented from lower to higher values by the color scale. SHAP values describe model behavior and should not be interpreted as measures of statistical significance or causal effects.

Table 4 summarizes the factors with the greatest relative importance in the random forest, gradient boosting, and artificial neural network models, while Figure 4 presents the corresponding SHAP-based interpretation of the SVM model. Across the random forest, gradient boosting, and ANN models, age was consistently the most influential feature, accounting for 33.10%, 41.10%, and 47.59% of the relative importance, respectively. Age was also among the factors with the greatest influence on the SVM predictions, indicating a consistent pattern across the different modeling approaches.

The number of chronic conditions also contributed meaningfully across models. Having one chronic condition accounted for 19.92% of the relative importance in random forest, 17.08% in gradient boosting, and 11.14% in the ANN, while having two chronic conditions was also prominent in the random forest and gradient boosting models. Similarly, the SHAP analysis indicated that the chronic-condition burden influenced the predictions generated by the SVM.

The number of previous COVID-19 infections was particularly influential in the random forest and ANN models, especially the category of two previous infections. By contrast, the SVM SHAP results placed greater emphasis on sex, age, time since the most recent infection, chronic conditions, and BMI. Within the fitted SVM model, female sex, older age, a longer time since the most recent infection, the presence of chronic conditions, and higher BMI generally shifted predictions toward the PCC category.

Overall, age and chronic-condition burden emerged as the most consistent influential factors across the algorithms, whereas the importance of sex, BMI, marital status, time since infection, and number of previous infections varied by model. Because the feature-importance percentages and SHAP values are calculated differently, they should not be compared as equivalent effect sizes. These findings describe how the models generated their predictions and should not be interpreted as statistically significant, independent, or causal associations. Given the limited discriminative performance of the models, the feature-importance and SHAP findings should be considered exploratory and require confirmation in larger, externally validated datasets.

## 4. Discussion

This study aimed to assess the prevalence of PCC in the capital of Jordan, investigate the common persistent symptoms after COVID-19, and employ machine-learning techniques to identify factors associated with PCC. Among the 963 community-based individuals with positive PCR results, 209 had PCC (22%). The prevalence we found in our study (22%) aligns with the reported prevalence in the literature, in which PCC rates have been reported to affect approximately 10% to 30% [18,19]. This alignment ensures the representation of the sample used in this study and highlights the PCC burden on public health, especially in low- to middle-income countries.

Our results showed that the most common persistent symptoms after COVID-19 were fatigue (45%), followed by bone/joint/muscle pain (34%), one neurological symptom (24%), one pulmonary/respiratory symptom (23%), and one mental health symptom (16%). These results are consistent with those reported by the WHO and other studies [20,21,22]. The involvement of the neurological, musculoskeletal, and pulmonary systems, besides fatigue and mental health, underscores the activity limitations and participation restrictions posed by PCC on individuals’ lives [23]. In addition, this highlights the importance of early detection of having PCC and the integral role of prompt responses from healthcare providers to prevent disability.

Primary unadjusted analysis of the study data comparing participants with and without PCC identified pre-existing chronic conditions and the number of prior COVID-19 infections as associated factors with PCC status. These two factors remained significant when multivariable modified Poisson regression was applied. Participants with exactly two chronic conditions had a significantly higher prevalence of PCC compared with those without chronic conditions. However, this association was not observed among participants with one or three or more chronic conditions. Therefore, the findings do not demonstrate a consistent dose–response relationship between the number of chronic conditions and PCC prevalence. The estimate for the category of three or more chronic conditions should also be interpreted cautiously given the relatively small number of participants in this group. This result may contribute to emerging evidence in the literature that suggests a higher risk of persistent symptoms post-COVID-19 in individuals with multiple chronic conditions [24]. This may be explained by the impaired immune responses, systemic inflammation, and diminished viral clearance that people with multiple chronic conditions usually suffer from, which is also the mechanism that was hypothesized to be associated with developing PCC [7,25]. These vulnerabilities may be exacerbated by the additional burden of several co-existing illnesses, increasing the likelihood that symptoms will persist. These results may suggest that people with multimorbidity are a high-priority category for early surveillance and focused rehabilitation therapies after acute COVID-19 infection.

Interestingly, we found a dose–response association between the risk of PCC and the number of prior COVID-19 infections. Compared with participants who had experienced a single infection, those with two infections had an approximately 88% higher prevalence of PCC, whereas participants with three or more infections had more than a threefold-higher prevalence. The increased risk of PCC with the number of previous infections may reflect individual susceptibility and vulnerability to COVID-19. This may also imply that recurrent exposure to the COVID-19 virus may gradually impede physiological recovery systems, possibly through mechanisms such as cumulative microbiome disturbance, immunological fatigue, mitochondrial dysfunction, or viral reservoir persistence [26,27]. Particularly notable was the stronger association observed among participants with three or more previous COVID-19 infections. These results have important implications for public health, emphasizing the necessity of continuing COVID-19 preventative measures, such as immunization and non-pharmacological therapies, even in people who have already recovered from infection. When assessing patients for functional deficits linked to PCC, physical therapists and rehabilitation specialists should pay close attention to reinfection history.

When machine-learning techniques were employed to evaluate the relative importance of these factors and their contributions to model predictions of PCC, the SHAP beeswarm plot indicated that female sex, age, longer time since last COVID-19 infection, number of chronic conditions, and higher BMI had the greatest influence on SVM predictions. These findings reflect the behavior of the fitted prediction model and should not be interpreted as evidence that these factors are independently associated with PCC. A study on the attributes of long COVID published in 2021 reported that female sex, BMI, and age were significant predictors of PCC [28]. A systematic review and meta-analysis on factors associated with PCC was published in 2023 [7]. This meta-analysis found that female sex, older age, higher BMI, smoking, pre-existing comorbidities, and previous hospitalization or ICU admission were risk factors significantly associated with developing PCC [7]. A multi-center study that was conducted on healthcare employees with PCC in the same year as our study (2022) found that the prevalence of PCC was 24%, with fatigue, myalgia, and cough being the most common persistent symptoms [21].

The findings for several variables differed between the regression and machine-learning analyses. In the complete-case modified Poisson model, age, sex, BMI, and time since the last COVID-19 infection were not significantly associated with PCC prevalence. In contrast, these variables were among the factors that contributed to the SVM predictions, with female sex, older age, higher BMI, and a longer time since the last infection generally shifting predictions toward the PCC category. These findings are not directly comparable, as modified Poisson regression estimates adjusted associations, whereas SHAP values describe how individual factors contribute to model predictions. Given the absence of statistically significant associations in the regression analysis and the limited discriminative performance of the machine-learning models, these variables should not be interpreted as independent associated factors for PCC.

Female sex has been reported frequently in the literature as a predictor of PCC, which may be explained by the fact that females are more susceptible to autoimmune disorders due to the effect of their hormones on viral binding [29,30]. Obesity and chronic conditions, such as diabetes, were found to be significant risk factors for severe COVID-19, hospitalization, and death [31]. Longer duration since the last COVID-19 infection also influenced predictions in the SVM model towards PCC, which can be explained by the fact that people need more time to recognize the persistent symptoms and relate them to the COVID-19 infection and report them to healthcare providers. This finding can also be interpreted as experiencing delayed symptom onset after the acute COVID-19 infection. Future longitudinal studies would be helpful in identifying the timing and development of PCC.

Another possible explanation is the documented association between female sex, pre-existing depression, and fatigue-related symptoms. Because many PCC symptoms are self-reported and inherently subjective, symptom reporting may be influenced by underlying psychological factors. Previous research has suggested that individuals with pre-existing mental health conditions may be more likely to report persistent symptoms following mild COVID-19, potentially increasing the likelihood of symptom over-reporting or misclassification rather than reflecting objective disease burden alone [22]. Similarly, the same study reported that men were less likely to report reduced work ability after mild COVID-19, suggesting that sex-related differences in symptom perception and reporting may contribute to the observed associations [22].

Our study is not without limitations. The cross-sectional, self-reported design does not establish attribution of symptoms to previous COVID-19 infection. Moreover, this study was conducted during the Omicron wave of the COVID-19 pandemic; therefore, the findings should be interpreted within the context of infections occurring during that period. Additionally, conducted at a single center in Jordan, the generalizability of results may be limited. However, the study was conducted at one of the main referral hospitals serving the population of the capital during the COVID-19 pandemic, providing a large and diverse patient population. While a large sample of adults with confirmed COVID-19 was included, post-COVID-19 symptoms were self-reported, introducing potential recall bias. Missing data on certain variables may impact statistical power, although the overall sample size maintained robustness. Despite these issues, the study offers valuable insights into PCC during the Omicron wave in a less represented region. An additional limitation relates to the validation of the machine-learning models. Model performance was evaluated using an internal training–testing split without external validation in an independent cohort or repeated cross-validation procedures.

## 5. Conclusions

The prevalence and associated factors of PCC were investigated in this study, and a relatively high percentage (22%) of participants were found to have persistent symptoms after infection. Fatigue was among the most common persistent symptoms, followed by musculoskeletal pain and other body systems. The results showed a significant public health burden and were consistent with previous research. Those with two pre-existing chronic diseases were more vulnerable to developing PCC. Prior COVID-19 infections showed a substantial dose–response association; those who had three or more infections were three times more likely to develop PCC. Female sex, greater BMI, the time since the previous infection, and the number of chronic conditions contributed the most in predicting PCC. The findings provide exploratory evidence regarding characteristics associated with PCC and factors contributing to model predictions. Given the limited discriminative performance of the machine-learning models and the absence of external validation, the models are not currently suitable for individual clinical prediction or decision-making. Further prospective studies and external validation are needed to determine whether these findings have clinical utility.

## Figures and Tables

**Table 1:** Participants’ characteristics and differences between individuals with PCC (*n* = 209) and without PCC (*n* = 754) (using Wilcoxon rank-sum test, Pearson’s Chi-squared test, and Fisher’s exact test).

Characteristic	Category or Statistic	All (*n* = 963)	No PCC (*n* = 754)	PCC (*n* = 209)	*p*-Value
Age	Mean years ± SD	47.4 ± 17.1	47.2 ± 17.5	45.5 ± 13.3	0.30
	Missing, *n*	241	196	45	
Sex	Female, *n* (%)	501 (52)	380 (50)	121 (58)	0.06
	Male, *n* (%)	462 (48)	374 (50)	88 (42)	
Marital Status	Married or engaged, *n* (%)	518 (75)	405 (75)	113 (77)	0.90
	Divorced/Widowed, *n* (%)	34 (4.9)	27 (5.0)	7 (4.8)	
	Single, *n* (%)	137 (20)	110 (20)	27 (18)	
	Missing, *n*	274	212	62	
Education Level	Degree above high school, *n* (%)	456 (67)	358 (67)	98 (67)	0.90
	High school or below, *n* (%)	229 (33)	180 (33)	49 (33)	
	Missing, *n*	278	216	62	
BMI	Mean ± SD	27.3 ± 5.0	27.2 ± 4.9	27.7 ± 5.3	0.50
	Missing, *n*	275	213	62	
Number of Medications	0, *n* (%)	359 (55)	286 (56)	73 (51)	0.70
	1, *n* (%)	127 (19)	99 (19)	28 (20)	
	2, *n* (%)	57 (8.7)	45 (8.7)	12 (8.5)	
	3 or more, *n* (%)	114 (17)	85 (17)	29 (20)	
	Missing, *n*	306	239	67	
Number of Chronic Conditions	0, *n* (%)	357 (52)	285 (53)	72 (49)	0.04
	1, *n* (%)	182 (27)	147 (27)	35 (24)	
	2, *n* (%)	91 (13)	61 (11)	30 (20)	
	3 or more, *n* (%)	56 (8.2)	46 (8.5)	10 (6.8)	
	Missing, *n*	277	215	62	
Time Since Last COVID-19 Event in Months	Mean months ± SD	17.6 ± 4.7	17.5 ± 4.7	17.9 ± 4.7	0.14
Number of COVID-19 Infections	1, *n* (%)	881 (91)	702 (93)	179 (86)	<0.01
	2, *n* (%)	64 (6.6)	43 (5.7)	21 (10)	
	3 or more, *n* (%)	18 (1.9)	9 (1.2)	9 (4.3)	
Number of Persistent Symptoms	1, *n* (%)	117 (12)	0 (0)	117 (12)	
	2, *n* (%)	51 (5.3)	0 (0)	51 (5.3)	
	3 or more, *n* (%)	41 (4.3)	0 (0)	41 (4.3)	
Fatigue	*n* (%)	95 (9.9)			
Bone/Joint/Muscle Pain	*n* (%)	71 (7.4)			
Urinary and Reproductive Symptoms	*n* (%)	3 (0.3)			
Number of Neurological Symptoms	0, *n* (%)	897 (93)			
	1, *n* (%)	50 (5.2)			
	2, *n* (%)	12 (1.2)			
	3 or more, *n* (%)	4 (0.4)			
Number of Pulmonary Symptoms	0, *n* (%)	888 (92)			
	1, *n* (%)	49 (5.1)			
	2, *n* (%)	13 (1.3)			
	3 or more, *n* (%)	13 (1.3)			
Number of Cardiac Symptoms	*n* (%)	16 (1.7)			
Number of Gastrointestinal Symptoms	*n* (%)	5 (0.5)			
Number of Mental Health Symptoms	0, *n* (%)	928 (96)			
	1, *n* (%)	33 (3.4)			
	2, *n* (%)	2 (0.2)			
Number of Endocrine Symptoms	*n* (%)	6 (0.6)			

**Table 2:** Adjusted prevalence ratios (PR) from the modified Poisson regression model.

Predictor	Adjusted PR [95% CI]	*p*-Value
Age (years)	0.9908 [0.9814, 1.0003]	0.0584
Sex—male	0.8758 [0.6294, 1.2186]	0.4313
Marital Status—divorced/widow	1.0579 [0.6757, 1.6563]	0.8057
Marital Status—single	1.3138 [0.5294, 3.2607]	0.5562
Education Level—highschool or below	1.0899 [0.7706, 1.5416]	0.6266
BMI	1.0104 [0.9748, 1.0473]	0.5732
Number of Chronic Conditions—1	0.9180 [0.6082, 1.3858]	0.6839
Number of Chronic Conditions—2	1.6849 [1.1203, 2.5342]	0.0122 *
Number of Chronic Conditions—3 or more	0.8275 [0.3771, 1.8159]	0.6367
Duration Since Last COVID-19 Infection (Months)	1.0081 [0.9750, 1.0424]	0.6349
Number of COVID-19 Infections—2	1.8823 [1.1585, 3.0584]	0.0107 *
Number of COVID-19 Infections—3 or more	3.0272 [1.7916, 5.1147]	<0.0010 *

* *p*-Value < 0.05.

**Table 3:** Performance metrics of all machine-learning models.

	AUROC (95% CI)	Accuracy	Recall (Sensitivity)	Specificity	Precision (PPV)	NPV	F1-Score
Logistic Regression	0.614 [0.507, 0.712]	0.549 [0.482, 0.617]	0.619 [0.474, 0.759]	0.530 [0.454, 0.606]	0.268 [0.183, 0.357]	0.833 [0.758, 0.901]	0.562 [0.527, 0.598]
Random Forest	0.551 [0.446, 0.647]	0.513 [0.440, 0.585]	0.524 [0.375, 0.675]	0.510 [0.435, 0.592]	0.229 [0.151, 0.319]	0.794 [0.710, 0.871]	0.659 [0.637, 0.681]
Gradient Boosting	0.582 [0.478, 0.680]	0.549 [0.477, 0.622]	0.452 [0.308, 0.609]	0.576 [0.490, 0.653]	0.229 [0.146, 0.329]	0.791 [0.713, 0.861]	0.678 [0.656, 0.700]
Support Vector Machines	0.615 [0.514, 0.713]	0.466 [0.399, 0.534]	0.690 [0.545, 0.825]	0.404 [0.327, 0.483]	0.244 [0.165, 0.322]	0.824 [0.736, 0.903]	0.635 [0.595, 0.675]
Artificial Neural Network	0.576 [0.480, 0.667]	0.451 [0.383, 0.523]	0.643 [0.500, 0.781]	0.397 [0.322, 0.476]	0.229 [0.156, 0.306]	0.800 [0.711, 0.889]	0.605 [0.564, 0.645]

PPV: positive predictive value. NPV: negative predictive value.

**Table 4:** Summary of factors’ importance across all algorithms.

Model	Leading Factors by Relative Importance
Random Forest	Age (33.10%); 1 chronic condition (19.92%); 2 chronic conditions (17.52%); 2 infections (9.91%)
Gradient Boosting	Age (41.10%); 1 chronic condition (17.08%); 2 chronic conditions (13.30%); married/engaged status (8.04%)
Artificial Neural Network	Age (47.59%); 2 infections (20.57%); single status (14.36%); 1 chronic condition (11.14%)

## Data Availability

Research data are available to interested researchers upon request from the corresponding author.

## Supplementary Materials

The following supporting information can be downloaded at: https://www.mdpi.com/article/10.3390/medicina62091739/s1.

## Appendix A

**Table A1:** Sensitivity analysis of adjusted prevalence ratios using modified Poisson regression with imputed data.

Predictor	Adjusted PR [95% CI]	*p*-Value
Age (years)	0.9965 [0.9898, 1.0033]	0.3094
Gender—male	0.8324 [0.6532, 1.0609]	0.1382
Marital Status—divorced/widow	1.0246 [0.5137, 2.0439]	0.945
Marital Status—single	0.8713 [0.4036, 1.8811]	0.7257
Education Level—high school or below	0.9931 [0.7392, 1.3341]	0.9632
BMI	1.0047 [1.0034, 1.0060]	<0.001 *
Number of Chronic Conditions—1	0.9100 [0.6473, 1.2792]	0.5872
Number of Chronic Conditions—2	1.5782 [1.1288, 2.2064]	0.0076 *
Number of Chronic Conditions—3 or more	0.8583 [0.4684, 1.5727]	0.6209
Duration of Last COVID-19 Infection (Months)	1.0222 [0.9971, 1.0479]	0.084
Number of COVID-19 Infections—2	1.6446 [1.1280, 2.3978]	0.0097 *
Number of COVID-19 Infections—3 or more	2.3643 [1.4591, 3.8311]	<0.001 *

* *p*-Value < 0.05, PR: prevalence ratios.
